# Idiopathic pericardial effusion in patients with hypertrophic cardiomyopathy

**DOI:** 10.1007/s10554-021-02424-8

**Published:** 2021-10-12

**Authors:** Sarinya Puwanant, Veraprapas Kittipibul, Nattakorn Songsirisuk, Sakun Santisukwongchote, Patita Sitticharoenchai, Pairoj Chattranukulchai, Sudarat Satitthummanid, Smonporn Boonyaratvej

**Affiliations:** 1 Division of Cardiovascular Medicine, Department of Medicine, Faculty of Medicine, Chulalongkorn University, Bangkok, Thailand; 2 Department of Pathology, Faculty of Medicine, Chulalongkorn University, Bangkok, Thailand; 3 Cardiac Center, King Chulalongkorn Memorial Hospital, Thai Red Cross Society, Bangkok, 10330 Thailand

**Keywords:** Hypertrophic cardiomyopathy, Pericardial effusion, Pathology

## Abstract

The aims of this study were to examine the prevalence of moderate to large (moderate–large) idiopathic pericardial effusion (i-PEF) in patients with hypertrophic cardiomyopathy (HCM) and to identify clinical and echocardiographic hemodynamic profiles associated with pericardial effusion. A total of 292 adult patients with HCM were studied. Fifteen patients with a history of factors associated with pericardial effusion including myocardial infarction, heart surgery or cardiac procedure within the last 12 months, autoimmune disease, hydralazine use, chronic kidney disease stage 3–4, tuberculosis, and malignancy were excluded. Of 277 eligible patients with HCM, 11 patients (4%) with moderate-large i-PEF were identified. Clinical tamponade was present in 1 patient. Compared to patients with HCM who had no or small pericardial effusion, patients with moderate-large i-PEF were younger and more likely to have right ventricular (RV) hypertrophy and reverse septal curvature. These patients also exhibited a greater maximal septal thickness, mean and systolic pulmonary pressure, and right atrial pressure (p < 0.05 for all). Pericardial fluid analysis and histopathological exams were performed in 7 and 3 patients, respectively. All examinations revealed transudative and nonspecific etiology of pericardial effusion. Idiopathic pericardial effusion and cardiac tamponade in patients with HCM was uncommon. The pathophysiology involved in pericardial effusion remains undetermined. Patients with moderate-large i-PEF frequently exhibited a phenotype of pulmonary hypertension and RV pressure overload.

**Supplementary Information:**

The online version contains supplementary material available at 10.1007/s10554-021-02424-8.

## Introduction

Pericardial effusion is a common disorder in clinical practice [1–3]. The common etiologies of pericardial effusion include infection, malignancy, connective tissue disease, immune process, myopericarditis, uremic, hypothyroidism, hydropericardium syndrome, or hemopericardium syndrome [1–3]. Recently, we observed patients with hypertrophic cardiomyopathy (HCM) who presented with moderate to large pericardial effusion of unknown etiology. The prevalence and clinical significance of pericardial effusion in patients with hypertrophic cardiomyopathy (HCM) has not been widely investigated. The aims of this study were to examine the prevalence of idiopathic pericardial effusion in patients with HCM and to identify clinical and echocardiographic characteristics associated with moderate to large pericardial effusion.

## Methods

### Study patients

The study protocol was approved by the Chulalongkorn University Institutional Review Board (IRB). A total of 292 adult (≥ 18 years old) patients with HCM who were referred for evaluation in the heart clinic at a tertiary center were reviewed for enrollment. The main reasons for referral were to establish the diagnosis and to consider septal reductive therapy. Patients with HCM diagnosed by family screening of an index case were not included. The diagnosis of HCM was based on a maximal left ventricular wall thickness ≥ 15 mm in one or more myocardial segments or ≥ 13 mm with a family history of HCM in the absence of other conditions associated with ventricular hypertrophy. Myocardial wall thickness was assessed by two-dimensional transthoracic echocardiography and/or cardiac magnetic resonance imaging (MRI) by standard technique [4]. Patients with a history of myocardial infarction within the last 12 months (n = 8), heart surgery or ablative procedure prior to cardiac imaging study (n = 1), autoimmune disease (n = 2), hydralazine use (n = 1), acute pericarditis/myocarditis (n = 0), tuberculosis (n = 1), malignancy (n = 1), human immunodeficiency viral (HIV) infection (n = 0), trauma (n = 0), radiation (n = 0), and chronic kidney disease stage 3–4 (n = 1) were excluded. A total of 277 patients were included in the study.

### Echocardiography

Comprehensive echocardiogram was performed in all patients using commercially available ultrasound machines, Vivid 7 GE-Vingmed (Milwaukee, WI), IE-33 Philips (Philips Medical System, Andover, Mass), and ProSound Alpha 10 *(*Hitachi Aloka Medical. Ltd., Tokyo, Japan*).* Respiratory variation of echocardiographic parameters was assessed by respirometer during echocardiographic examination in patients with pericardial effusion ≥ 2 cm or suspicion of cardiac tamponade. Echocardiographic images were digitally stored in EchoPAC and QLAB software package for off-line analysis. Asymmetrical septal hypertrophy (ASH) was defined as septal-to-free-wall ratio of ≥ 1.3 [4]. Apical HCM including pure and mixed apical HCM (apical/septal) was defined as previously described [5, 6]. Septal morphology subtypes were classified as sigmoid, reverse-curve, neutral, and apical variant as previously described [7]. Pericardial effusion, an echo-free space visualized between parietal and visceral pericardium at end diastole, was semi-quantitatively classified as trivial (present in only systole), small (< 1 cm), moderate (1–2 cm), large (> 2 cm), or very large/massive (> 2.5 cm) [2]. Right ventricular (RV) systolic and diastolic echocardiographic parameters were assessed according to the guidelines for echocardiographic assessment of the right heart in adults endorsed by the EAE and the Canadian Society of Echocardiography [8]. Pulmonary arterial systolic pressure in the absence of pulmonary stenosis was estimated by the peak continuous-wave Doppler of the tricuspid regurgitation velocity with 4V^2^ plus right atrial pressure estimated from inferior vena caval (IVC) size and its collapsibility [8–10]. Mean pulmonary arterial pressure was estimated by the peak continuous-wave Doppler of the pulmonary regurgitation velocity as 4V^2^ plus right atrial pressure estimated from IVC size and its collapsibility [11]. Pulmonary hypertension (PH) was defined as estimated pulmonary arterial systolic pressure > 35 mmHg.

### Pathological examination

Pericardial and myocardial pathological specimens were fixed in formalin and paraffin embedded in patients who underwent pericardial or endomyocardial biopsy or surgical myectomy. The surgical specimens of pericardium and myocardium were stained with hematoxylin and eosin and Movat pentachrome. Pericardial specimens were additionally stained with acid fast bacilli (AFB) and modified AFB and cultured for aerobe, anaerobic, tuberculosis, and fungal organisms. Gross pericardial specimens were measured for maximal thickness. Pericardial histopathological slides were reviewed by an expert cardiac pathologist for the presence of calcification, fibrosis, inflammation, caseous and non-caseous granulomas, mesothelial abnormalities, hemosiderin deposition, and malignancy. Myocardial histopathological slides were examined for myocyte hypertrophy and disarray, dysplastic intramural coronary arterioles with medial and intimal thickening, and fibrosis. Myocardial specimens were additionally stained for Congo red and periodic acid-Schiff to exclude cardiac amyloidosis and glycogen storage disease. Surgical myectomy and endomyocardial biopsy were performed in 34 and 2 patients, respectively. Myocardial histopathological specimens in 36 patients confirmed HCM.

### Statistical analysis

Categorical data are presented as frequency and percentage. Continuous data are expressed as mean ± standard deviation (SD). Differences in means were compared by Student’s t test for variables with normal distribution and Wilcoxon–rank sum test for variables with non-normal distribution. Categorical variables were compared using chi’s square test or Fischer’s exact test, where appropriate. Due to small numbers of patients with moderate and large pericardial effusion, multivariate analysis was not performed. A *p* value < 0.05 was considered significant.

## Results

### Clinical and echocardiographic characteristics

**Fig. 1:**
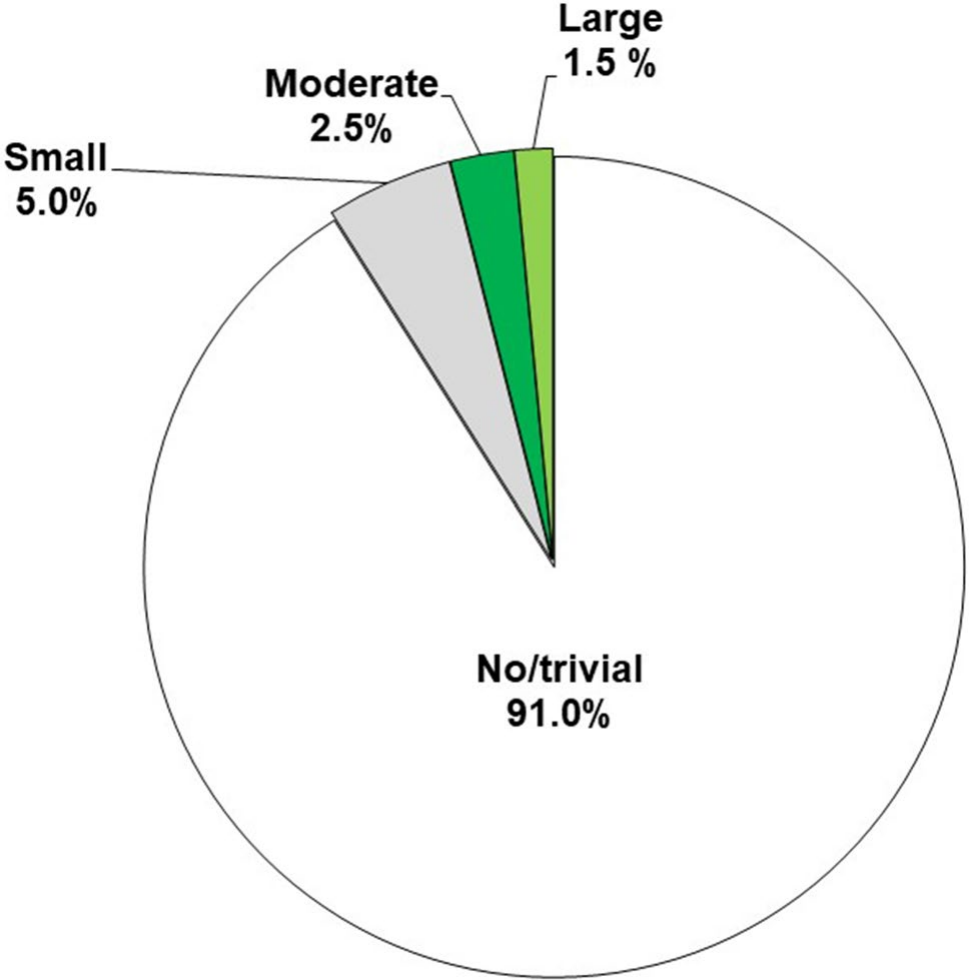
The prevalence of idiopathic pericardial effusion in patients with HCM

**Fig. 2:**
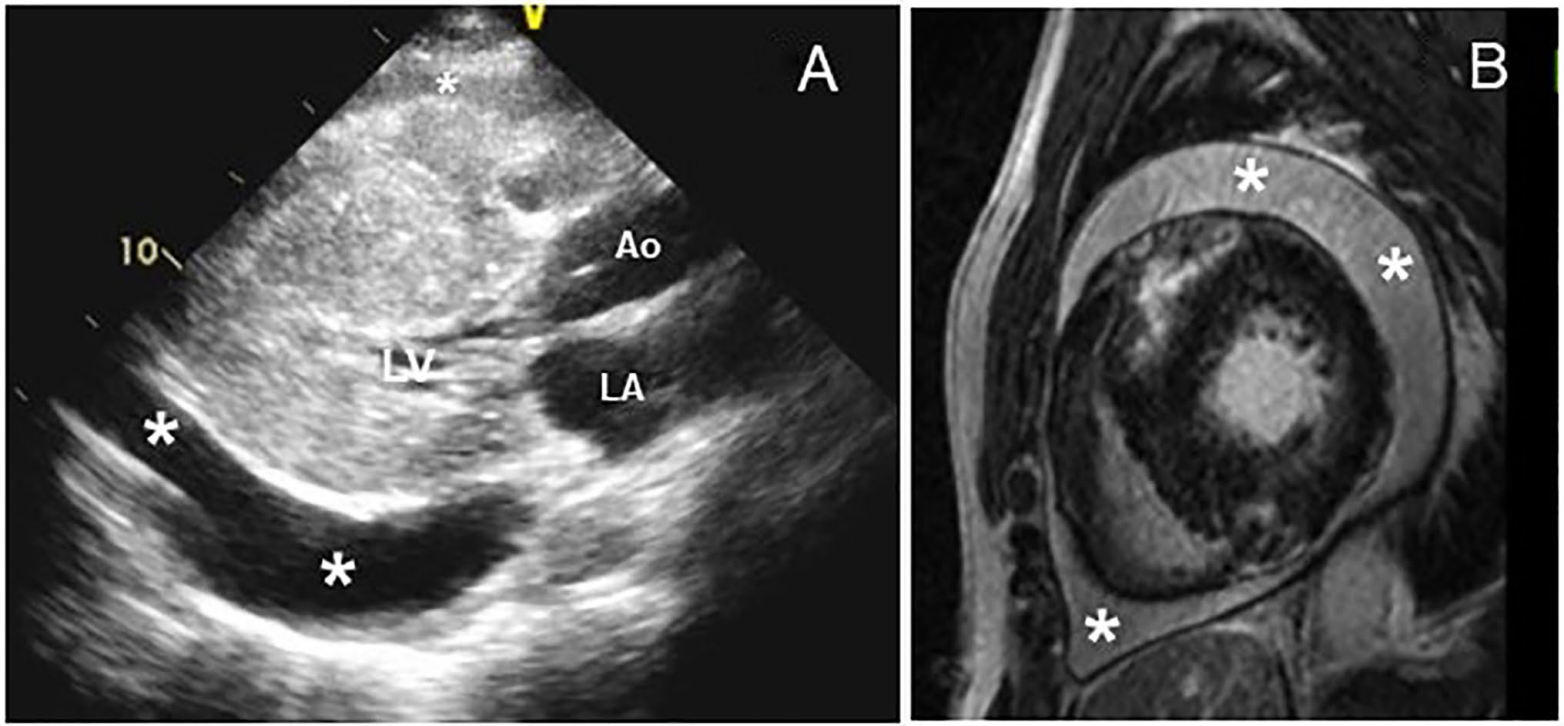
A massive circumferential pericardial effusion (asterisks) in a 40-year-old man with a hypertrophic cardiomyopathy (patient #2) demonstrated by transthoracic echocardiogram (**a**) and cardiovascular magnetic resonance imaging (**b**)

Among the 277 eligible patients with HCM, 11 patients (4%) with moderate to large idiopathic pericardial effusion were identified. Moderate and large pericardial effusion was found in 7 and 4 patients, respectively. Clinical tamponade was present in 1 patient, while echocardiographic tamponade was present in 2 patients. An additional 14 (5%) patients exhibited small pericardial effusion (Fig. 1). Baseline characteristics of patients are shown in Table 1. Compared to patients with HCM who had no or small pericardial effusion, patients with moderate to large idiopathic pericardial effusion were younger (49 ± 16 vs. 63 ± 16 years; p = 0.01). Significant clinical differences included being more likely to have pulmonary hypertension (90% vs. 40%; p < 0.01) and reverse septal curvature (72% vs. 29%; p = 0.02), a greater maximal septal thickness (24 ± 5 vs. 18 ± 5 mm.; p < 0.01), higher RV free wall thickness (10 ± 2 vs. 8 ± 3 mm; p < 0.01), higher mean pulmonary pressure (29 ± 5 vs. 22 ± 6 mmHg; p < 0.01), higher systolic pulmonary pressure (48 ± 11 vs. 36 ± 11 mmHg; p < 0.01), and higher right atrial pressure (15 ± 5 vs. 6 ± 4 mm; p < 0.01). Figure 2A and video A illustrate a large pericardial effusion identified on transthoracic echocardiogram in a patient with HCM (Patient #4 in Table 2). Figure 2B illustrates a massive circumferential pericardial effusion and normal pericardial findings identified on cardiovascular magnetic resonance imaging in the same patient (Fig. 2B).

**Table 1:** Clinical and echocardiographic characteristics by presence or absence of moderate to large pericardial effusion

	All (n = 277)	Moderate to large pericardial effusion (n = 11)	Non-significant pericardial effusion (n = 266)	p value
Age	63 ± 16	49 ± 16	63 ± 16	0.01*
Female	161 (58%)	6 (55%)	155 (58%)	0.81
NYHA Class III–IV [n (%)]	48 (17%)	2 (18%)	46 (18%)	0.16
Systolic blood pressure (mmHg)	130 ± 19	131 ± 20	130 ± 19	0.88
Diastolic blood pressure (mmHg)	74 ± 11	74 ± 6	75 ± 11	0.50
Atrial fibrillation [n (%)]	36 (13%)	1 (9%)	35 (13%)	0.69
Major Phenotype [n (%)]				
Asymmetrical septal hypertrophy	138 (50%)	8 (73%)	130 (49%)	0.68
Pure apical	56 (20%)	2 (18%)	54 (20%)	
Mixed apical	30 (11%)	0	30 (12%)	
Concentric	47 (17%)	1 (9%)	46 (17%)	
Localized/mid	6 (2%)	0	6 (2%)	
Reverse-curve septal morphology	86 (31%)	8 (72%)	78 (29%)	0.02*
Large pericardial effusion	4 (1%)	4 (36%)	0	< 0.01*
Beta blocker [n (%)]	121 (73%)	11 (100%)	197 (74%)	0.05
Calcium channel blocker [n (%)]	39 (14%)	1 (9%)	38 (14%)	0.62
Septal myectomy [n (%)]	34 (12%)	6 (54%)	28 (10%)	< 0.01*
Alcohol septal ablation [n (%)]	2 (1%)	0 (0%)	2 (1%)	0.77
Maximal septal thickness (mm)	19 ± 5	24 ± 5	18 ± 5	< 0.01*
Resting LVOT gradient > 30 mmHg [n (%)]	60 (36%)	3 (33%)	57 (35%)	0.88
LVEDD (mm)	43 ± 8	41 ± 9	43 ± 8	0.77
LVEF (%)	71 ± 12	71 ± 13	71 ± 12	0.88
LAVI (ml/m^2^)	39 ± 16	39 ± 13	39 ± 17	0.99
RAVI (ml/m^2^)	33 ± 16	40 ± 24	33 ± 15	0.42
RV free wall thickness (mm)	9 ± 3	10.3 ± 2.0	8.4 ± 2.7	0.01*
Estimated RAP (mmHg)	7 ± 4	15 ± 5	6 ± 4	< 0.01*
Estimated pulmonary arterial systolic pressure (mmHg)	36 ± 11	48 ± 11	36 ± 11	< 0.01*
Estimated mean pulmonary arterial pressure (mmHg)	22 ± 6	29 ± 5	22 ± 6	< 0.01*
Pulmonary hypertension (n,%)	117 (42%)	10 (90%)	107 (40%)	< 0.01*
TAPSE (mm)	17.9 ± 4.6	18.3 ± 4.3	18.0 ± 4.6	0.76

*LV* left ventricular; *LVEDD* left ventricular end diastolic diameter; *LVEF* Left ventricular ejection fraction; *LAVI* left atrial volume index; *LVEDD* left ventricular end diastolic diameter; *LVOT* left ventricular outflow tract; *mm* millimeter; *NYHA* New York Heart Association; *RAP* right atrial pressure; *RAVI* right atrial volume index; *RV* right ventricular; *TAPSE* tricuspid annular plane excursion

**Table 2:** Clinical and cardiac imaging characteristics of HCM patients with pericardial effusion

No	Age/gender	Effusion (mm)	Effusion	EF (%)	RV free wall thickness (mm)	Maximal thickness	Resting LVOT gradient	Phenotype	RAP (mmHg)	RVSP (mmHg)	ANA and RF	CMV IgM	Anti-HIV Ab	Pathological Exam	Pericardial fluid color	Pericardial fluid type	Pericardial fluid PCR for TB	Pericardial fluid culture for aerobe, TB, and fungus	Pericardial fluid cytology	Pericardial thickness by cardiac MRI
1	57 F	17	Moderate	84	9.4	32	19	ASH	18	44	Neg	Neg	Neg	Not performed	Straw, clear	Transudate	Neg	Neg	Neg	Normal
2	71 M	36	Large	73	9.0	19	20	ASH	20	72	Neg	Neg	Neg	Normal	Straw, clear	Transudate	Neg	Neg	Neg	N/A
3	24 F	13	Moderate	87	11.3	19	63	ASH	8	54	Neg	Neg	Neg	Not performed	N/A	N/A	N/A	N/A	N/A	Normal
4	40 M	30	Large	90	13.0	31	40	ASH	20	39	Neg	Neg	Neg	Normal	Straw, clear	Transudate	Neg	Neg	Neg	Normal
5	54 M	12	Moderate	70	9.1	21	12	Apical	10	31	Neg	Neg	Neg	Not performed	N/A	N/A	Neg	N/A	N/A	Normal
6	56 M	26	Large	76	7.0	30	18	Apical	15	49	Neg	Neg	Neg	Not performed	Straw, clear	Transudate	Neg	Neg	Neg	Normal
7	51 M	14	Moderate	65	9.2	22	23	Concentric	20	42	Neg	N/A	Neg	Not performed	N/A	N/A	Neg	N/A	N/A	Normal
8	21 F	13	Moderate	45	10.0	21	15	ASH	15	51	Neg	Neg	Neg	Not performed	N/A	N/A	N/A	N/A	N/A	Normal
9	47 F	23	Large	61	13.1	29	14	ASH	15	58	Neg	Neg	Neg	Normal	Straw, clear	Transudate	Neg	Neg	Neg	Normal
10	52 F	15	Moderate	68	12.0	20	25	ASH	15	53	Neg	Neg	Neg	Not performed	Straw, clear	Transudate	Neg	Neg	Neg	Normal
11	67 M	16	Moderate	65	11.0	22	66	ASH	3	39	N/A	Neg	Neg	Not performed	Straw, clear	Transudate	Neg	Neg	Neg	Normal

*ANA* antinuclear antibody; *ASH* asymmetrical septal hypertrophy; *CMV* cytomegalovirus; *EF* ejection fraction; *F* female; *HIV* Human Immunodeficiency Virus; *LVOT* left ventricular outflow tract; *M* male; *mm* millimeter; *MRI* magnetic resonance imaging; *N/A* not applicable; *Neg* negative; *RAP* right atrial pressure; *RF* rheumatoid factor; *RV* right ventricular; *RVSP* right ventricular systolic pressure; *S* systolic forward flow; *TB* tuberculosis

### Pericardial fluid analysis and pathological examination

**Fig. 3:**
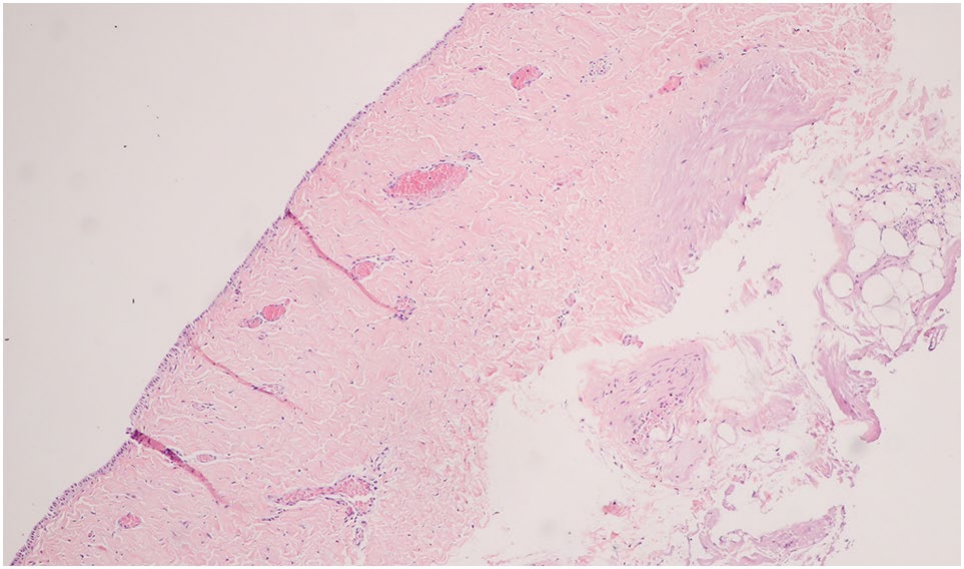
Example of pericardial histopathological findings of a 40-year-old patient who underwent surgical myectomy and pericardial biopsy. The pericardium revealed normal pericardial thickening and intact mesothelial cells with no active inflammation, granuloma, malignancy, or calcification

Table 2 shows clinical, cardiac imaging, and pericardial characteristics in patients with moderate to large pericardial effusion. None of these patients had a clinical syndrome of active viral bacterial, parasitic infection, mononucleosis syndrome, autoimmune disease, or antiphospholipid syndrome. Thyroid stimulating hormone levels were considered normal in all patients. Additionally, acute pericarditis diagnosed by the European Society of Cardiology (ESC) criteria [12] was not evident in any patient. Among those with large pericardial effusion, pericardial histopathological exams were performed in 3 patients with massive pericardial effusion. Pericardial biopsies were performed at the time of surgical myectomy in one patient and at the time of subxiphoid pericardial window in other patients with a large pericardial effusion where echocardiography-guided pericardiocentesis was deemed to be unsuccessful (< 1.2 cm in diastole adjacent to the right ventricle). Findings of these patients revealed normal pericardial thickening with no active inflammation. Mesothelial cells were intact. No granuloma, malignancy or calcification was visualized. Pericardial fluid analysis was performed in 7 patients with moderate to large pericardial effusion with all revealed as transudative. Pericardial-fluid and tissue stains for AFB and modified AFB were all negative for tuberculosis and nocardia. Pericardial fluid and tissue cultures for aerobe, anaerobe, and fungus were negative. Pericardial fluid and tissue cultures and polymerase chain reaction (PCR) for tuberculosis were also negative. Pericardial fluid cytology was negative for malignancy. Figure 3 illustrates pathological findings of pericardium in patient #4 (Table 2) who underwent surgical myectomy and pericardial biopsy.

## Discussion

This study is the first to examine the prevalence of idiopathic pericardial effusion among those with HCM and the clinical and pericardial pathological profiles of these patients. The major findings of the study are: (1) the prevalence of moderate to large pericardial effusion in patients with HCM was 4% (11/277); (2) pericardial pathological and fluid analysis in patients with massive pericardial effusion were characterized by normal pericardial thickening, nonspecific histological findings, and transudative profile with no evidence of infectious or inflammatory process, or autoimmune or inflammatory reactive etiology; and (3) patients with moderate to large pericardial effusion were more likely to have pulmonary hypertension (PH), elevated right atrial pressure, right ventricular hypertrophy and septal hypertrophy.

The normal pericardial sac contains 20–50 ml of pericardial fluid [1, 2]. A pericardial effusion occurs when excess pericardial fluid accumulates in the pericardial sac [1, 2]. Pericardial fluid is normally generated by plasma ultrainfiltrate and drains to the mediastinal, tracheobronchial, peri-esophageal and pleural lymphatic systems [2]. The excessive pericardial fluid is typically caused by (1) increased production of pericardial fluid following infectious or noninfectious inflammatory pericardial process (mostly exudate), (2) impaired reabsorption or drainage of pericardial fluid (transudate) including heart failure or PH, (3) systemic conditions including hypoalbuminemia or hypothyroidism (transudate/hydropericardium), or (4) conditions associated with cardiac and great-vessel injuries (hemopericardium) [2, 3, 13]. In our study, the prevalence of moderate to large pericardial effusion in patients with HCM was uncommon (4% of patients with HCM). We found that no inflammatory, infectious, or specified etiologies were identified in these patients. Patients with moderate to large pericardial effusion did have higher estimated right atrial and pulmonary arterial pressures compared to those with no or small pericardial effusion. The pathogenesis of pericardial effusion in PH is currently unclear. Previous studies have reported 15–65% of patients with PH had pericardial effusion [13–15]. Hinderliter et al. demonstrated that severity of RV dysfunction is associated with pericardial effusion in patients with PH, and among invasive intracardiac and pulmonary hemodynamic indices, mean right atrial pressure correlated best with the size of pericardial effusion [16]. Fröhlich et al. suggested that venous and/or lymphatic congestion may be involved in the etiology of pericardial effusion in heart failure. [15]Further, they proposed that cytokines released in severe heart failure may play a role in the instigation of pericardial effusion by way of systemic inflammatory inducing capillary leakage which increase production of pericardial effusion. Ong et al. reported that 38% of patients with HCM had PH. In our study, 42% of overall patients with HCM and 90% of patients with moderate to large pericardial effusion had PH [17]. Whether pericardial effusion was coincident or associated with HCM and PH remains to be determined.

The low prevalence of pericardial effusion led to limited statistical power to find significant differences in most of the studied covariates, limiting inferences regarding possible etiologies and mechanisms. Further study to clarify the association of pericardial effusion and right ventricular dysfunction or PH in patients with HCM is required.

To our best knowledge, there has been no systematic review or published data about idiopathic pericardial effusion or tamponade in HCM patients. This study is the first to describe the clinical and pericardial pathological profiles in an HCM cohort. The severity of pericardial effusion along with the severity of right atrial and pulmonary pressures in patients with pericardial pathological confirmation confirms a similar trend of findings among the entire cohort.

### Study limitations

Pericardial biopsy or pericardiocentesis was not performed in all patients with pericardial effusion. Traditionally, patients with HCM with small to moderate pericardial effusion with no clinical tamponade do not require invasive biopsy or pericardiocentesis. Simultaneous invasive pulmonary pressure and vascular resistance measurements were not performed in all patients with pericardial effusion and PH. Doppler interrogation of tricuspid regurgitation to estimate peak pulmonary arterial systolic pressure has been validated and widely accepted and remains the best noninvasive measure available [9, 10]. Another study limitation is that antibodies to Epstein-Barr, Coxsackie, Influenza, Herpes, or hepatitis viruses were not tested in all patients. They were only tested in patients who were clinically suspicious or uncertain for these viral infections. However, those patients did not have a clinical syndrome of viral infection, mononucleosis, bacterial or parasitic infection, autoimmune disease, antiphospholipid syndrome, or acute pericarditis. Additionally, all pericardial analyses revealed transudative profiles making viral associated pericardial effusion unlikely.

## Conclusions

Idiopathic moderate to large pericardial effusion was found uncommon and occurred in only 4% of patients with HCM. All patients with a completed pericardial fluid analysis showed transudative profiles. Whether pericardial effusion was coincident or associated with HCM remains undetermined. Since patients with moderate to large pericardial effusion exhibited greater septal thickness, pulmonary pressure, and RV free wall thickness, we suggest that PH may be involved in the pathophysiology of pericardial effusion in patients with HCM.

## Supplementary Information

Below is the link to the electronic supplementary material.

Supplementary file1 (MP4 11365 kb)
